# High prevalence of anal high‐risk HPV infection among transwomen: estimates from a Brazilian RDS study

**DOI:** 10.1002/jia2.25691

**Published:** 2021-03-27

**Authors:** Emilia M Jalil, Erin C Wilson, Laylla Monteiro, Luciane S de Velasque, Ana Cristina G Ferreira, Sandro C Nazer, Ruth K Friedman, Valdilea G Veloso, José Eduardo Levi, Beatriz Grinsztejn

**Affiliations:** ^1^ National Institute of Infectious Diseases FIOCRUZ Rio de Janeiro Brazil; ^2^ San Francisco Department of Public Health San Francisco CA USA; ^3^ Mathematics and Statistics Department UNIRIO Rio de Janeiro Brazil; ^4^ Tropical Medicine Institute USP São Paulo Brazil

**Keywords:** prevalence, papillomavirus infections, transgender persons, sexually transmitted disease, anal cancer

## Abstract

**Introduction:**

As the leading sexually transmitted infection worldwide, human papillomavirus (HPV) may disproportionately affect transwomen. We aimed to estimate anal HPV prevalence, especially focusing on high‐risk (hr)‐HPV types and evaluate their associated factors among transwomen living in Rio de Janeiro, Brazil.

**Methods:**

Transwomen enrolled in a respondent‐driven sampling (RDS)‐based survey conducted between August 2015 and January 2016 self‐collected anal samples, which were promptly stored at minus 80°C. After DNA extraction, HPV detection and genotyping were performed using the PapilloCheck test. We estimated HPV prevalences and evaluated the correlates of anal hr‐HPV infection using a regression logistic model.

**Results:**

Out of 345 transwomen, 272 (78.8%) were included in this analysis (122 [44.9%] HIV‐positive). No participant had ever received HPV vaccination. Among participants enrolled, 212 (77.9%) were positive for any anal HPV type and 165 (60.7%) for hr‐HPV. Most common hr‐HPV were as follows: HPV16 (17.6%), HPV68 (14.7%), HPV39 (14.3%), HPV56 (12.5%), HPV51 (11.4%) and HPV52 (11.0%). HIV‐positive transwomen had three times the odds of having an hr‐HPV compared to HIV‐negative transwomen. Participants who had a current rectal Neisseria gonorrhoeae infection had 3.7 times the odds of being coinfected with hr‐HPV. Among HIV‐positive transwomen, neither antiretroviral therapy use, undetectable viral load, current and nadir CD4 counts were associated with anal hr‐HPV infection.

**Conclusions:**

Brazilian transwomen in our study exhibit some of the highest population‐specific rates of HPV and hr‐HPV. There is an urgent need to elucidate the burden of HPV infection, prevalence of HPV‐related diseases and access to and uptake of HPV vaccination among transwomen, especially from low‐ and middle‐income settings.

## 1 INTRODUCTION

Human papillomavirus (HPV) is the leading sexually transmitted infection (STI) worldwide. Most sexually active people become infected with HPV at some point in their lives [1, 2, 3]. HPV types with an oncogenic potential are grouped as high‐risk HPV (hr‐HPV). HPV is a ubiquitous pathogen in the anogenital region of cisgender (i.e. non‐transgender) individuals. The global cervical HPV prevalence is 11% to 12% among cisgender women without cytological abnormalities [4, 5]; there has been a strong association between cervical and anal hr‐HPV [6]. Nevertheless, it is higher among groups disproportionally impacted by STI, such as cisgender men who have sex with men (MSM) [7, 8].

Besides a necessary factor to cervical oncogenesis [9], hr‐HPV infection is also a causal factor of other cancers [10, 11]. Almost 90% of anal cancers are attributable to HPV, particularly HPV16, which is the most carcinogenic type in the anus [6, 12]. Although a rare outcome, there has been an increasing trend in anal cancer incidence worldwide [13]. This increase may be linked to a change in sexual behaviour effecting HPV transmission [14]. Anal cancer is more common among people living with HIV (PLHIV), a population growing in size as treatments continue to increase lifespan [15, 16, 17].

Transgender women (henceforth “transwomen”) experience numerous socio‐economic factors that increase their vulnerability to HIV/STIs [18]. However, very little data exist on HPV infection among transwomen. A recent meta‐analysis identified only four studies on anal HPV infection in Brazil, none among transwomen [8]. We aimed to estimate anal HPV and hr‐HPV prevalences and to evaluate their associated factors among transwomen living in Rio de Janeiro, Brazil.

## 2 METHODS

This is a secondary analysis of *Transcender*, a cross‐sectional study that enrolled 345 transwomen using respondent‐driven sampling (RDS) at the Evandro Chagas National Institute of Infectious Diseases (INI)‐FIOCRUZ, Rio de Janeiro, Brazil, between August 2015 and January 2016. RDS has been used to obtain robust and diverse samples of hard‐to‐reach populations [19]. Procedures have been described previously [20]. Briefly, eligible participants self‐identified as transwomen, lived in Rio de Janeiro or metropolitan area, and were aged 18+ years. We selected 12 diverse seed participants in our formative phase to ensure that the sample did not over‐represent groups by age, HIV status, or sex work. Participants recruited up to five peers until the target sample size was reached and the sample composition from one wave to the next differed by less than 2% (equilibrium).

We offered HIV/STIs testing to all participants. A trained staff member provided anal self‐collection materials and explained how to perform the procedure using illustrated instructions [21]. In a private bathroom dedicated only for self‐collection, participants were instructed to insert a sterile brush (Kolplast, Brazil) 3 to 4 cm into the anus in a comfortable position, to circle it three times, and to remove the brush and place it in a ThinPrep vial (18ml). Participants immediately disposed of their vials in a sealable plastic bag and delivered them to a team member. Samples were promptly stored at minus 80°C and shipped on dry ice to the Virology Laboratory at the *Instituto de Medicina Tropical da Universidade de São Paulo*. Analysis methods have been described elsewhere [22]. In summary, we used the QIAamp DNA Mini Kit (Qiagen, Chattlesworh, CA) for DNA extraction. HPV detection and genotyping used the PapilloCheck HPV‐Screening Test (Greiner Bio‐One GmbH, Frickenhausen, Germany), which detects 15 hour‐HPV (16, 18, 31, 33, 35, 39, 45, 51, 52, 56, 58, 59, 68, 73, 82), two probable hr‐HPV genotypes (53 and 66) and seven low‐risk (lr)‐HPV genotypes (6, 11, 40, 42, 43, 44, 70).

Syphilis screening used Venereal Disease Research Laboratory (VDRL) tests, and positive results were confirmed by a microhaemagglutination assay for *Treponema pallidum*. We detected rectal *Chlamydia trachomatis* (CT) and *Neisseria gonorrhoeae* (NG) infection by using the Abbott Real Time platform and the Amplification Reagent Kit (Abbott Molecular, Des Plains, IL, USA) in INI‐FIOCRUZ Laboratory. Indeterminate results were repeated with the same tests on the same samples. All participants living with HIV collected CD4+ count (Becton Dickinson FACScan) and HIV viral load (VL). Previous CD4+ counts were retrieved from the Brazilian National Registry, which records most measures performed in the country. Participants self‐reported antiretroviral therapy (ART) use.

We estimated prevalence of any anal HPV, at least one HPV type in the 2‐valent (HPV16, 18), 4‐valent (HPV6, 11, 16, 18) and 9‐valent (HPV6, 11, 16, 18, 31, 33, 45, 52, 58) vaccines, hr‐HPV (positive for 1+ hour‐HPV detected by the test) and individual HPV types.

Continuous variables were reclassified as categorical. We defined active or recent syphilis as VDRL titers of at least 1/8 plus a positive microhaemagglutination assay for *T. pallidum*. The nadir CD4+ count was defined as the participant’s lowest level ever recorded.

We used RDS‐unadjusted data. Descriptive analysis used median and interquartile range (IQR) and absolute and relative frequencies. We compared participants according to HIV status using chi‐square tests. We used a regression logistic model to evaluate the correlates of anal hr‐HPV infection. Variables with *p* < 0.2 in the univariable logistic regression analysis and potential confounders were included in the initial multivariable model. A backward elimination procedure guided the removal of non‐significant variables until we reached a final multivariable model where all variables had a *p* ≤ 0.05 or modified the effect of other variables. Statistical analysis was performed using R software version 3.4.3.

This study was approved by the INI‐FIOCRUZ Institutional Review Board. All participants provided an informed consent form. This study received grants of the Brazilian Research Council (CNPq) and the National Institute of Allergy and Infectious Diseases (NIAID‐NIH).

## 3 RESULTS AND DISCUSSION

Among 345 transwomen enrolled in the *Transcender* study, 341 (98.8%) agreed to and conducted anal specimen collection, 69 (20.0%) had invalid anal samples due to insufficient material and 272 (78.8%) had samples with valid results and were included in the present analysis. HIV‐negative status was more common among participants with invalid samples than those with valid samples (72.9% vs. 55.1% respectively). Overall, participants were aged 30 years (IQR 24 to 37), 76.8% were non‐white, and 48.9% were currently on hormones. Only 16 (5.9%) participants had gender‐affirming surgery. Ever and currently doing sex work were reported by 79.0% and 48.9% respectively. No participant had received HPV vaccine. HIV‐infection was present in 122 individuals (44.9%). Among those living with HIV, 83 were on ART, 43.7% were virologically suppressed, and median current and nadir CD4 count were, respectively, 631 cells/mm^3^ (IQR 384 to 833) and 380 cells/mm^3^ (IQR 196 to 576).

Out of 272 participants tested for anal HPV, 212 (77.9%) and 165 (60.7%) were positive for any HPV and hr‐HPV respectively (Table 1).

**Table 1:** Prevalence of anal HPV types, overall and according to HIV status, among transwomen enrolled in the *Transcender* study, Rio de Janeiro, 2015 to 2016

HPV type	Overall (N = 272)	HIV‐positive (N = 122)	HIV‐negative (N = 150)	*p*‐value
Any HPV type^a^	212 (77.9)	105 (86.1)	107 (71.3)	**<0.01**
Any 2‐valent vaccine types^b^	61 (22.4)	38 (31.1)	23 (15.3)	**<0.01**
Any 4‐valent vaccine types^c^	101 (37.1)	55 (45.1)	46 (30.7)	**0.01**
Any 9‐valent vaccine types^d^	139 (51.1)	81 (66.4)	58 (38.7)	**<0.001**
Multiple HPV types^e^	153 (72.2)	90 (85.7)	63 (58.9)	**<0.001**
hr‐HPV^f^				
Any hr‐HPV types	165 (60.7)	92 (75.4)	73 (48.7)	**<0.001**
Multiple hr‐HPV types^g^	96 (58.2)	66 (71.7)	30 (41.1)	**<0.001**
HPV16	48 (17.6)	27 (22.1)	21 (14.0)	0.08
HPV18	16 (5.9)	14 (11.5)	2 (1.3)	**<0.001**
HPV31	16 (5.9)	12 (9.8)	4 (2.7)	**0.01**
HPV33	22 (8.1)	14 (11.5)	8 (5.3)	0.07
HPV35	17 (6.2)	14 (11.5)	3 (2.0)	**<0.01**
HPV39	39 (14.3)	26 (21.3)	13 (8.7)	**<0.01**
HPV45	14 (5.1)	11 (9.0)	3 (2.0)	**<0.01**
HPV51	31 (11.4)	17 (13.9)	14 (9.3)	0.24
HPV52	30 (11.0)	17 (13.9)	13 (8.7)	0.17
HPV56	34 (12.5)	22 (18.0)	12 (8.0)	**0.01**
HPV58	29 (10.7)	23 (18.9)	6 (4.0)	**<0.001**
HPV59	25 (9.2)	18 (14.8)	7 (4.7)	**<0.01**
HPV68	40 (14.7)	23 (18.9)	17 (11.3)	0.08
HPV73	9 (3.3)	9 (7.4)	0 (0.0)	**<0.001**
HPV82	28 (10.3)	19 (15.6)	9 (6.0)	**0.01**
lr‐HPV				
HPV6	43 (15.8)	21 (17.2)	22 (14.7)	0.57
HPV11	21 (7.7)	11 (9.0)	10 (6.7)	0.47
HPV40	18 (6.6)	11 (9.0)	7 (4.7)	0.15
HPV42	42 (15.4)	27 (22.1)	15 (10.0)	**0.01**
HPV43	16 (5.9)	7 (5.7)	9 (6.0)	0.93
HPV44	64 (23.5)	39 (32.0)	25 (16.7)	**<0.01**
HPV53^h^	35 (12.9)	24 (19.7)	11 (7.3)	**<0.01**
HPV55	64 (23.5)	39 (32.0)	25 (16.7)	**<0.01**
HPV66^h^	26 (9.6)	14 (11.5)	12 (8.0)	0.33
HPV70	24 (8.8)	16 (13.1)	8 (5.3)	**0.02**

HIV, human immunodeficiency virus; HPV, human papillomavirus; hr‐HPV, high‐risk HPV; lr‐HPV, low‐risk HPV.

^a^ Positive for 1 + HPV type

^b^ HPV 16 or 18

^c^ HPV 6, 11, 16 or 18

^d^ HPV 6, 11, 16, 18, 31, 33, 45, 52 or 58

^e^ more than 1 HPV type (analysis restricted to those HPV positive, N = 212)

^f^ HPV 16, 18, 31, 33, 35, 39, 45, 51, 52, 56, 58, 59, 68, 73, 82

^g^ more than 1 hr‐HPV (denominator restricted to hr‐HPV‐positive, N = 165)

^h^ HPV 53 and 66 are currently considered as probable high‐risk types.

The most common hr‐HPV was HPV16 (17.6%). Only three people had both HPV16 and 18 (1.1%), and none were positive for all 4‐ and 9‐valent vaccine types. Transwomen living with HIV had a significantly higher prevalence of most types of anal HPV than those HIV negative. HPV6 and HPV43 had decreasing prevalences according to age (Table 2). Transwomen aged 25+ years had higher prevalences of HPV44 and HPV55 as compared to those aged 18 to 24 years, whereas HPV56 had a lower prevalence among transwomen aged ≥35 years.

**Table 2:** Prevalence of anal HPV types according to age among transwomen enrolled in the *Transcender* study, Rio de Janeiro, 2015 to 2016

	18 to 24 years (N,%)	25 to 35 years (N,%)	>35 years (N,%)	*p*‐value
HPV6	18 (24.7)	18 (15.4)	7 (8.5)	**0.02**
HPV11	7 (9.6)	9 (7.7)	5 (6.1)	0.72
HPV16	13 (17.8)	24 (20.5)	11 (13.4)	0.43
HPV18	6 (8.2)	6 (5.1)	4 (4.9)	0.66
HPV31	6 (8.2)	6 (5.1)	4 (4.9)	0.70
HPV33	7 (9.6)	11 (9.4)	4 (4.9)	0.44
HPV35	5 (6.8)	8 (6.8)	4 (4.9)	0.83
HPV39	13 (17.8)	14 (12)	12 (14.6)	0.53
HPV40	6 (8.2)	5 (4.3)	7 (8.5)	0.40
HPV42	15 (20.5)	20 (17.1)	7 (8.5)	0.10
HPV43	7 (9.6)	9 (7.7)	0 (0)	**<0.01**
HPV44	10 (13.7)	34 (29.1)	20 (24.4)	**0.05**
HPV45	2 (2.7)	5 (4.3)	7 (8.5)	0.23
HPV51	11 (15.1)	13 (11.1)	7 (8.5)	0.44
HPV52	7 (9.6)	13 (11.1)	10 (12.2)	0.87
HPV53	10 (13.7)	12 (10.3)	13 (15.9)	0.49
HPV55	10 (13.7)	34 (29.1)	20 (24.4)	**0.05**
HPV56	11 (15.1)	19 (16.2)	4 (4.9)	**0.04**
HPV58	4 (5.5)	12 (10.3)	13 (15.9)	0.11
HPV59	6 (8.2)	13 (11.1)	6 (7.3)	0.62
HPV66	9 (12.3)	12 (10.3)	5 (6.1)	0.40
HPV68	15 (20.5)	17 (14.5)	8 (9.8)	0.17
HPV70	5 (6.8)	14 (12)	5 (6.1)	0.28
HPV73	1 (1.4)	6 (5.1)	2 (2.4)	0.44
HPV82	8 (11)	15 (12.8)	5 (6.1)	0.3

HPV, human papillomavirus.

Younger participants had significantly higher odds of anal hr‐HPV infection (Table 3). Transwomen living with HIV had three times the odds of anal hr‐HPV infection compared to those HIV‐negative. Participants with a rectal NG infection had 3.9 times the odds of hr‐HPV coinfection. Among transwomen living with HIV, ART use, undetectable VL and CD4+ counts were not associated with anal hr‐HPV infection.

**Table 3:** Correlates of anal hr‐HPV infection among transwomen in *Transcender* study, Rio de Janeiro, 2015 to 2016

Characteristic	hr‐HPV‐positive (N = 165), %	Univariable		Multivariable	
		Crude OR	*p*‐value	Adjusted OR	*p*‐value
Age^a^					
18 to 24	48 (65.8)	1.5 (0.8‐ 2.9)	0.22	2.4 (1.1 to 5.2)	0.03
25 to 35	71 (60.7)	1.2 (0.7 to 2.1)	0.52	1.9 (1.0 to 3.8)	0.06
>35	46 (56.1)	1		1	
Race/color					
Black	38 (58.5)	1			
Mixed/other	95 (66.0)	1.4 (0.8 to 2.5)	0.30		
White	32 (50.8)	0.7 (0.4 to 1.5)	0.38		
Monthly income^a^,^b^					
≤U$130	66 (58.4)	1			
U$131 to 260	51 (62.2)	1.1 (0.6 to 2.0)	0.59		
>U$260	34 (59.6)	1.2 (0.7 to 2.1)	0.88		
Years of schooling^a^					
<4	9 (52.9)	1			
4 to 8	57 (67.1)	1.8 (0.6 to 5.2)	0.27		
>8	99 (58.2)	1.2 (0.4 to 3.4)	0.67		
Smoking					
Never	51 (58.6)	1			
Former	22 (51.2)	0.7 (0.4 to 1.5)	0.42		
Current	92 (64.8)	1.3 (0.7 to 2.2)	0.35		
Binge drinking^c^					
No	55 (59.8)	1			
Yes	110 (61.1)	1.1 (0.6 to 1.8)	0.83		
Any illicit drug use in the last 12 months					
No	64 (56.6)	1			
Yes	101 (63.5)	1.3 (0.8 to 2.2)	0.25		
Current hormone use					
No	89 (64.0)	1			
Yes	76 (57.1)	0.7 (0.5 to 1.2)	0.25		
Previous reassignment surgery					
No	159 (62.1)	1			
Yes	6 (37.5)	0.4 (0.1 to 1.0)	0.06		
Sex work					
Never	35 (61.4)	1			
Former	44 (53.7)	0.7 (0.4 to 1.4)	0.37		
Currently	86 (64.7)	1.2 (0.6 to 2.2)	0.67		
N. male sex partners in the last six months^a^					
0 to 4	38 (54.3)	1			
5 to 9	15 (60.0)	1.3 (0.5 to 3.3)	0.62		
10+	103 (64.0)	1.5 (0.8 to 2.6)	0.17		
Condomless anal sex with last 3 partners					
No	48 (67.6)	1		1	
Yes	102 (57.0)	0.6 (0.4 to 1.1)	0.12	0.6 (0.3 to 1.2)	0.16
Condomless anal sex with main partner					
No	54 (58.1)	1			
Yes	104 (61.5)	1.2 (0.7 to 1.9)	0.58		
Consistent condom use					
No	132 (60.6)	1			
Yes	26 (59.1)	0.9 (0.5 to 1.8)	0.86		
Self‐reported condyloma					
No	140 (60.3)	1			
Yes	25 (64.1)	1.2 (0.6 to 2.4)	0.66		
HIV status					
Negative	73 (48.7)	1		1	
Positive	92 (75.4)	3.2 (1.9 to 5.5)	<0.001	4.0 (2.2 to 7.3)	**<0.001**
Current active syphilis					
No	115 (60.5)	1			
Yes	48 (60.0)	1.0 (0.6 to 1.7)	0.94		
Current rectal CT					
Negative	134 (59.8)	1			
Positive	25 (64.1)	1.2 (0.6 to 2.5)	0.61		
Current rectal NG					
Negative	141 (58.3)	1		1	
Positive	18 (81.8)	3.2 (1.2 to 11.4)	0.04	3.9 (1.3 to 14.4)	**0.02**

CT, *Chlamydia trachomatis*; hr‐HPV, high‐risk human papillomavirus; NG, *Neisseria gonorrhoeae*; OR, odds ratio.

^a^ Continuous variables were reclassified as categorical

^b^ US$1.00 = R$3.85

^c^ defined as six or more alcoholic drinks on any occasion.

These data add to the limited research on anal HPV infection among transwomen. In the United States, unvaccinated transwomen had a significantly higher anal HPV prevalence than unvaccinated MSM (88.6% vs. 70.9%), even after stratifying by HIV status [23]. Argentinian transwomen who were sex workers had an extremely high anal HPV prevalence (97.4%) [24], as well as Peruvian transwomen in general (95.6%) [25]. In contrast, an Italian clinical study observed that only 38.2% of trans people (13/34) had any anal HPV, though the small sample limits generalizability [26].

Rates of anal HPV and hr‐HPV in our sample of transwomen are higher than previously described among individuals with diverse gender identities and sexual orientations. A meta‐analysis found that anal HPV prevalences varied widely between cisgender men (mostly MSM) and women [6]. Their data identified any anal HPV in 24.6% out of 12,097 cisgender women, with a higher estimate among PLHIV (43.9%) than in HIV‐negative participants (18.7%) [6]. The HIM Study described that 16.3% of all men (MSM and men who have sex with women [MWSW]) had anal HPV. A Brazilian meta‐analysis enrolling female and male participants with low‐ and high‐risk behaviour identified lower rates of anal HPV than in our study (any HPV: 25.7%, hr‐HPV: 14.1%) [8].

Anal hr‐HPV detection was significantly higher among younger transwomen. There is a strong relationship between age and cervical HPV prevalence [27]. Nevertheless, this association is conflicting in the anus. Previous studies in MSM demonstrated high, stable anal HPV prevalence across different age groups. An US study observed that 26% of 1,400 MSM had any anal hr‐HPV with no difference in age groups [28]. Among Australian MSM, there was no consistent trend in HPV prevalence with increasing age [29]. A Chinese study on 578 MSM (50 with HIV) identified a decreasing trend in anal HPV prevalence with increasing age: 71% (≤19 years), 62% (20 to 29 years), 64% (30 to 39 years) and 54% (40+ years) [30]. Nevertheless, Nyitray et al. identified decreasing age‐specific anal HPV prevalence with the highest rates among MSM and MWSW aged 18 to 24 years in three countries, including Brazil [31]. Finally, contrasting findings observed that anal HPV prevalence increased from 24.5% among Chinese MSM aged ≤19 years to 55.8% among those aged 40+ years [32].

Transwomen living with HIV had a higher HPV and hr‐HPV prevalences compared to HIV‐negative participants, consistent with previous findings. MSM have the highest population‐specific HPV rates reported to date, especially among those living with HIV [31, 33, 34]. Any anal HPV prevalence was higher among MSM with HIV (81%) as compared to HIV‐negative MSM (47%), MWSW living with HIV (44%) and HIV‐negative MWSW (12%) [34]. Anal HPV prevalence among MSM was 4 to 10 times higher than among MWSW [31]. A meta‐analysis identified substantially higher pooled prevalence of any anal HPV and hr‐HPV in MSM living with HIV (92.6% and 73.5%) than in those HIV‐negative (63.9% and 37.2%) [33].

The 4‐valent HPV vaccine, available at no cost in Brazil since 2014, is currently offered to girls aged 9 to 14 years, boys aged 9 to 14 years and people living with HIV aged 9 to 26 years (regardless of gender identity) [35]. Nevertheless, HPV vaccine coverage in the country is still very low. The cumulative vaccine coverage (two‐dose course) was 45.1% in females between 2014 and 2017, and only 20.2% of the targeted male population received at least one dose in 2017 [36]. There are no Brazilian published data on HPV vaccine uptake according to HIV status, nor other gender identities. In Canada, HPV vaccine uptake was low among male individuals living with HIV, including MSM and those with low socio‐economic status [37]. In Brazil, a highly discriminatory setting hinders sexual/gender minorities from accessing healthcare services [38, 39]. Transphobia in Brazilian healthcare services has been previously described as the most prominent barrier to reduced willingness to seek HIV prevention or healthcare in general [39]. Although HPV‐related diseases occur independently of sociodemographic characteristics, their distribution is unequal among individuals with different socio‐economic status [40, 41]. As a highly disadvantaged group, transwomen bear synergistic vulnerabilities that may ultimately lead to low vaccine uptake and disproportionate risk for anal HPV‐related lesions.

This study has several limitations. First, although RDS survey is commonly used for hard‐to‐reach populations, its results may not be generalizable. In addition, the cross‐sectional design does not allow us to establish causal inference. We also did not evaluate anal cytology or histopathology data, which could ultimately reinforce the burden of anal HPV infection among transwomen. Syphilis definition only included laboratory results and did not consider clinical data. Finally, the high proportion of invalid samples may be due to user error. Two rectal samples had to be collected (for HPV and CT/NG), which may create burden on participants and interfered with the process. Although self‐collection has been described as an acceptable tool, including for anal samples, the proportion of invalid samples varied from 5% up to 37% across studies [21, 24, 42, 43]. Transwomen without HPV results (refusal or invalid samples) more frequently were HIV‐negative, which may have led to an overestimation of HPV prevalence among transwomen living with HIV. As PLHIV commonly access health services, they may be more familiar with performing laboratory exams including self‐collection, which could have skewed results for this group. Despite limitations, our data fill an important gap in the limited information on HPV among transwomen and is consistent with other studies showing high HPV prevalence among this population.

## 4 CONCLUSIONS

Our extremely high rates of HPV and hr‐HPV among transwomen show that this group is disproportionately affected by HPV and may be at a high risk for cancer. HPV16 was the most prevalent anal hr‐HPV type. Prevalence of hr‐HPV was higher among younger participants, those living with HIV and concomitant rectal NG infection. There is an urgent need to elucidate the burden of HPV infection and to increase access to and uptake of HPV vaccination among transwomen, especially from low‐ and middle‐income settings.

## COMPETING INTERESTS

The authors declare no conflict of interests.

## AUTHORS’ CONTRIBUTIONS

BG, EMJ, ECW and VGV conceived the analysis and interpreted the findings. BG, EMJ, ECW and VGV drafted the manuscript. LSV and EMJ performed the statistical analyses. JEL supervised the biological analysis, interpreted the results and provided biological inputs. EMJ, LM, LSV and ACFG helped with data acquisition, interpretation of the results and drafting the manuscript. All authors read and approved the final manuscript.
